# Uncoupling of Influenza A Virus Transcription and Replication through Mutation of the Unpaired Adenosine in the Viral RNA Promoter

**DOI:** 10.1128/JVI.00636-13

**Published:** 2013-09

**Authors:** Aartjan J. W. te Velthuis, Lauren Turrell, Frank T. Vreede, Ervin Fodor

**Affiliations:** University of Oxford, Sir William Dunn School of Pathology, Oxford, United Kingdom

## Abstract

Transcription and replication of the influenza A virus RNA genome are mediated by the viral RNA polymerase from a promoter consisting of the partially base-paired 3′ and 5′ termini of viral genome segments. Here we show that transcription and replication can be uncoupled by mutation of an unpaired adenosine in the 5′ strand of the promoter. This residue is important for transcription but not replication by being essential for the cap-binding activity of the RNA polymerase.

## TEXT

During the replication cycle of the influenza A virus, the viral RNA-dependent RNA polymerase (RdRp), a complex consisting of the PA, PB1, and PB2 subunits, replicates and transcribes the eight segments of negative sense viral RNA (vRNA) that make up the viral genome (1, 2). Both replication and transcription require the catalytic residues in the RdRp domain of PB1 for the condensation of nucleotides. However, whereas replication can start *de novo*, transcription initiation is dependent on a capped RNA primer and involves a process in which (i) the PB2 subunit binds the 5′ cap of host pre mRNAs, (ii) the bound host pre-mRNAs are cleaved 9 to 15 nucleotides downstream of the cap by the PA subunit to yield capped primers, (iii) the primers are aligned to the 3′ end of the genome segments, and (iv) PB1 catalyzes nucleotide addition to the 3′-OH end of the primer.

**Fig 1:**
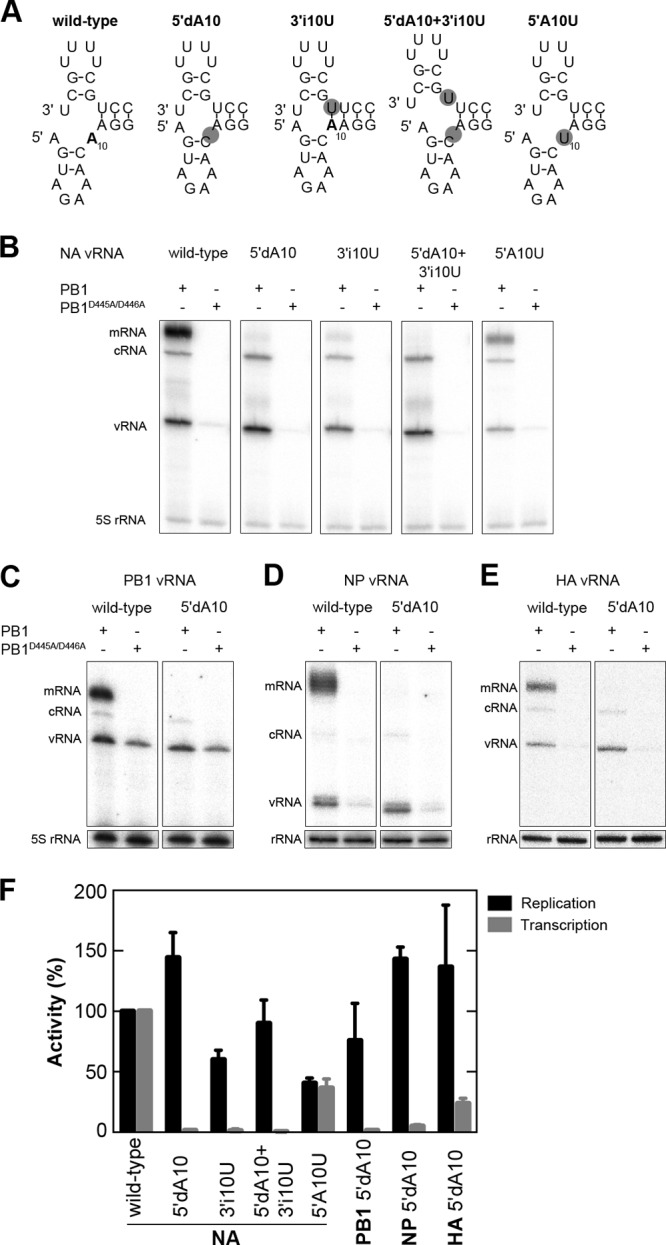
Effects of vRNA promoter mutations on transcription and replication by the viral RdRp. (A) Schematic representation of wild-type and mutant influenza A virus promoter structures according to the corkscrew model. Residue 5′A10 is in boldface, and mutated residues are shaded in gray. (B) Primer extension analysis was performed to assess the RNA levels derived from wild-type and 5′dA10, 3′i10U, 5′dA10 plus 3′i10U, and 5′A10U mutant templates. The RNA species that are produced during replication (vRNA and cRNA) and transcription (mRNA) are indicated. 5S rRNA is shown as a loading control. (C to E) Primer extension analysis (24) to assess the RNA levels produced from wild-type and 5′dA10 mutant PB1-, NP-, and HA-encoding vRNAs. (F) Graph showing the mean RNA levels of three independent experiments. Error bars represent standard deviations (*n* = 3).

Replication and transcription by the viral RdRp are both initiated from a vRNA promoter that consists of the partially base-paired 3′ and 5′ termini of viral genome segments (3–6). Upon association with the RdRp, this promoter is believed to fold into a corkscrew-like conformation (Fig. 1A, wild type) (7), in which various elements have been shown to be crucial for RdRp activity, including the chemical properties of nucleosides, intrastrand loops, and interstrand base pairs (7–9). The function of an unpaired adenosine residue at position 10 of the 5′ strand (5′A10) (10, 11), however, remains largely unresolved. Replacement of 5′A10 with uridine (5′A10U) does not dramatically reduce promoter binding by the RdRp (4), while mutation of this residue to guanosine, uridine, or cytosine leads only to minor effects in endonuclease activity and reporter gene expression assays (7, 12, 13). In contrast, elimination of the unpaired 5′A10 by inserting a uridine residue into the 3′ end opposite 5′A10 or by replacing the resultant A-U base pair with G-C leads to abolishment of reporter gene expression (7, 14). Together these results suggest that it is not the chemical nature of 5′10A, but rather its effect on the overall promoter structure, that is critical for influenza A virus RNA synthesis.

To investigate the role of the unpaired 5′10A of the vRNA promoter in viral replication and transcription in more detail, we modified an authentic segment 6 (NA) vRNA-expressing plasmid (15) using site-directed mutagenesis by deletion of 5′10A. Next we transfected this plasmid into 293T cells together with plasmids expressing the RdRp subunits and NP of influenza A/WSN/33 virus (16) to reconstitute viral RNP (vRNP) complexes in cell culture. Forty-eight hours after transfection, the cells were harvested, the RNA was extracted using TRIzol, and the RNA levels were assessed using reverse transcription primed with ^32^P-labeled oligonucleotides (16, 17). As shown in Fig. 1B, the wild-type NA vRNA template was efficiently replicated and transcribed, as is evident from the increased vRNA, cRNA, and mRNA levels relative to a negative control where PB1 with mutations in the polymerase active site (17) was expressed. The 5′dA10 template demonstrated a dramatic loss of transcriptional activity but, surprisingly, supported efficient replication (Fig. 1B and F).

To address the question of whether the unpaired nature of 5′10A or the chemical properties of the residue are important for supporting viral transcription, we generated (i) a 3′ uridine insertion opposite 5′ A10 to enable its base pairing (3′iU10), (ii) a 5′dA10 and 3′i10U double mutation as a control for 3′iU10, and (iii) a 5′A10U substitution to control for the chemical properties of position 10 (Fig. 1A). As shown in Fig. 1B and F, mutants 3′iU10 and 3′i10U plus 5′d10A showed deleterious effects on transcription similar to those of 5′d10A, while mutant 5′A10U resulted in a relatively minor decrease in transcription and replication. To investigate further whether the unpaired nature of 5′10A is involved in the transcription of other genome segments, we deleted 5′A10 in segments 2 (PB1), 5 (NP), and 4 (HA). We introduced an internal stop codon in segments 2 and 5 to prevent additional PB1 or NP expression from mRNAs generated by the viral RdRp. As shown in Fig. 1C to F, the deletion of 5′A10 abolished transcription, but not replication, in the three other vRNA templates studied, implying that the presence of this residue is essential for the transcription for all viral segments.

**Fig 2:**
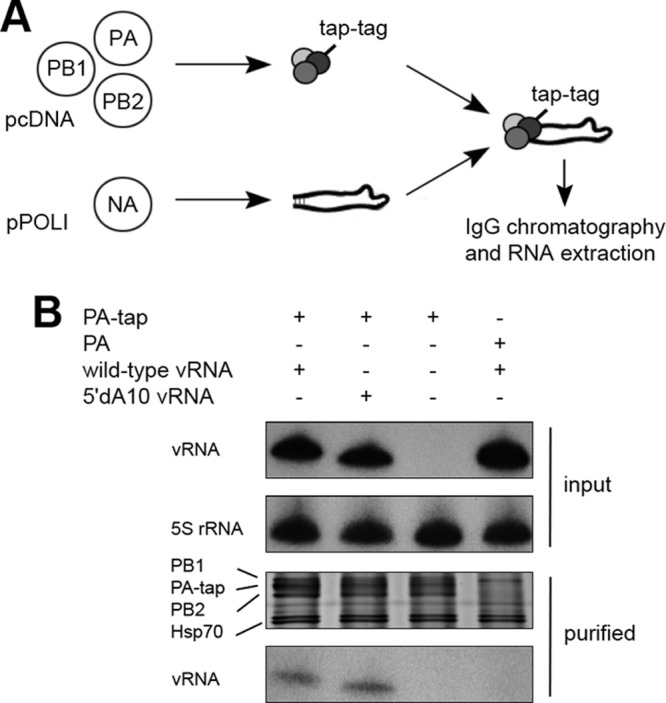
Binding of viral RdRp to vRNA templates containing wild-type or mutant 5′dA10 promoters. (A) Schematic representation of the “in cell culture” vRNA binding assay. Plasmids encoding NA vRNA templates were cotransfected with plasmids expressing TAP-tagged influenza A virus polymerase (PB1, PB2, and PA-tap). (B) Input RNA levels as analyzed by primer extension and autoradiography (top panel). Ribosomal 5S rRNA is shown as loading control (second panel). Partially purified viral polymerase was analyzed by SDS-PAGE and staining with silver (third panel). The RdRp subunits as well as the copurifying Hsp70 proteins are indicated. Levels of RNA copurified with RdRp are shown in the bottom panel.

Transcriptional activity of the viral RdRp on a vRNA template depends on the ability of the RdRp to bind the vRNA promoter and to bind and endonucleolytically cleave host pre-mRNAs to generate 5′ capped primers (1). To investigate the binding of the 5′dA10 mutant vRNA promoter by the viral RdRp *in vivo*, we transfected 293T cells with plasmids expressing PB1, PB2, tandem affinity purification (TAP)-tagged PA (18), and NA vRNA (Fig. 2A; note that NP was omitted to prevent replication and transcription). Subsequently, viral RdRp was purified from cell lysates using IgG-Sepharose chromatography (19), and the copurified RNA was isolated and quantitated using primer extension. As shown in Fig. 2, equal amounts of wild-type and 5′dA10 NA vRNA were detected (Fig. 2B), showing that wild-type and mutant templates were bound with equal efficiencies. No vRNA signals were present in the control samples containing an untagged PA subunit or no vRNA template (Fig. 2B). These data are in agreement with efficient replication of 5′dA10 NA vRNA by viral RdRp (Fig. 1B and F).

**Fig 3:**
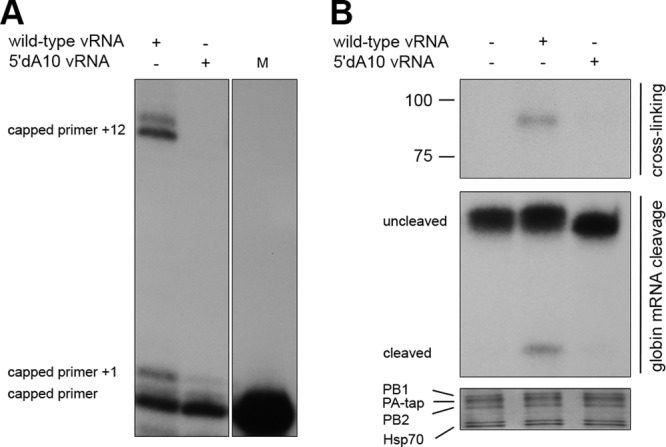
Effects of 5′10A deletion in the vRNA template on extension of a capped RNA primer, cap binding, and endonucleolytic activity of the viral RdRp. (A) ^32^P-labeled capped RNA was extended by RdRp in the presence of the wild-type or 5′10A deletion mutant vRNA template. The major product and the product extended by one nucleotide are indicated. The ^32^P-labeled capped RNA primer (M) is shown in the right panel. (B, top) ^32^P-labeled capped RNA was UV cross-linked to RdRp associated with wild-type, 5′10A deletion mutant, or no template vRNA. The cross-linked product is expected to migrate at ∼85 kDa. (Middle) Globin mRNA was used as a capped RNA substrate for RdRp associated with either wild-type, 5′10A deletion mutant, or no template vRNA. The slower-migrating bands correspond to the uncleaved globin mRNA, while the faster-migrating band represents cleaved globin mRNA. (Bottom) SDS-PAGE analysis of the RdRp. Labeling of the polymerase subunit bands is according to Fig. 2 and Deng et al. (18).

Since the binding of the RdRp to the mutant 5′dA10 NA vRNA template was unimpaired, we next investigated whether the 5′A10 deletion had an effect on 5′ cap binding, endonucleolytic cleavage of capped RNA, or the ability of the RdRp to extend a 5′ capped RNA primer. To test this, RdRp associated with wild-type or 5′dA10 mutant vRNAs (prepared as described for Fig. 2) was incubated with a ^32^P-radiolabeled and capped RNA oligonucleotide (16, 20) in the presence of 1 mM ATP, 0.5 mM CTP, and 0.5 mM GTP. Extension of the capped oligonucleotide in this assay starts at residue 3′G3 and is predicted to terminate after the addition of 12 nucleotides at residue 3′A15, due to the absence of UTP. As shown in Fig. 3A, the RdRp associated with wild-type NA vRNA produced a major product of the expected length and a longer minor product that was likely synthesized through misincorporation at position 3′A15. In addition, a single nucleotide addition was observed in the presence of the wild-type template, possibly due to nontemplated RdRp activity (16, 20). In contrast, no extended RNA products were detected with RdRp preparations that contained the 5′dA10 mutant vRNA (Fig. 3A). These results strongly suggest that 5′10A is important for cap binding by the RdRp, possibly by facilitating a conformational change in the RdRp. To address the role of 5′10A in cap binding, we performed an assay of the binding of RdRp to ^32^P-radiolabeled capped RNA using UV cross-linking. SDS-PAGE analysis of RdRp demonstrated that the RdRp preparations with the mutant template were impaired in their ability to bind capped RNA compared to those associated with the wild-type template (Fig. 3B). Impaired binding to capped RNA is predicted to result in impaired endonucleolytic cleavage of capped RNA. To test this, we incubated RdRp associated with wild-type or 5′10A deletion mutant NA vRNA with globin mRNA and analyzed cleavage by performing primer extension analysis of globin mRNA using primer 5′-GTGACCGCAGACTTCTCCTC-3′. We found that wild-type NA vRNA was able to produce a cleavage product, while no cleavage product was observed with the RdRp bound to the mutant NA vRNA, confirming that the reduced cap binding efficiency also resulted in an impaired capability of the purified RdRp to cleave capped mRNA (Fig. 3B).

In this study we sought to define the role of the unpaired adenosine residue at position 10 in the 5′ terminus of influenza A virus vRNAs. In contrast to the previously published hypothesis that 5′10A might be critically involved in promoter recognition and binding by the viral RdRp (10), our “in cell culture” vRNA promoter binding assay showed that recombinant polymerase binds a mutant vRNA template containing a 5′10A deletion as well as a wild-type template. Furthermore, we observed that vRNA templates with a 5′10A deletion are fully competent in supporting replication, an activity critically dependent on promoter recognition. This thus argues against a major role of this residue in vRNA recognition by the viral RdRp. However, templates with a 5′10A deletion or templates in which an inserted U residue in the 3′ arm of the promoter allowed base pairing of 5′10A were unable to support transcription. Analysis of the various steps in transcription initiation revealed that the 5′10A deletion affected the ability of the viral RdRp to bind 5′ capped RNAs, consequently blocking all downstream steps involved in transcription. This is in agreement with previous studies reporting inhibition of reporter gene expression as a result of such mutations.

In the absence of an influenza A virus RdRp structure, we can currently only speculate about the exact mode of action by which 5′A10 controls capped RNA binding by the RdRp without interfering with the ability of the RdRp to replicate RNAs. Deletion of 5′10A may result in an altered vRNA promoter structure, in which, for instance, base pair formation between 5′9C and 3′9G can efficiently compete with the formation of the 5′ and 3′ intrastrand base pairs, as proposed by the corkscrew model. Indeed, the formation of these 5′ intrastrand base pairs was previously shown to be critical for endonuclease activity of the RdRp (12, 13), suggesting that abolishment of the 5′ hairpin loop through the 5′A10 deletion could lead to the observed loss of transcriptional activity. Surprisingly, however, deletion of 5′10A does not affect replication, which implies that the processes of transcription and replication rely on fundamentally different vRNA promoter structures. It has been proposed that replication is carried out by a *trans*-acting RdRp that is not part of the vRNP structure (21). In addition, such *trans*-acting RdRps have been observed to depend on short viral RNAs (svRNAs), RNAs consisting of about 22 to 27 nucleotides (nt) of the 5′ end of vRNA, as cofactors (22, 23). Our data suggest that the deletion of 5′10A in these svRNAs does not interfere with their ability to promote RNA genome replication. Although the exact mode of action by which 5′A10 controls the RdRp remains thus unclear, we hope that our observations will facilitate and inspire further studies into the intriguing mechanisms regulating the transcriptional and replicative activities of the influenza A virus RNA polymerase on the vRNA template.
